# How do mothers with young children perceive endocrine-disrupting chemicals?: an exploratory qualitative study

**DOI:** 10.4069/kjwhn.2023.11.28

**Published:** 2023-12-28

**Authors:** SoMi Park, ChaeWeon Chung

**Affiliations:** 1 Wonju College of Nursing, Yonsei University, Wonju, Korea; 2 College of Nursing, Research Institute of Nursing Science, Seoul National University, Seoul, Korea

**Keywords:** Endocrine disruptors, Mothers, Perception, Qualitative research

## Abstract

**Purpose:**

Despite the health impacts of endocrine-disrupting chemicals (EDCs) beginning in the early stages of life, there is little research on the perception of EDCs among Korean mothers, who are primarily responsible for protecting children. This study aimed to explore how mothers with young children perceived EDCs for their concerns, the issues they faced, and the way they dealt with them.

**Methods:**

An exploratory qualitative design was utilized. Twelve mothers who were recruited from snowball sampling participated in voluntary interviews. Individual in-depth interviews lasting approximately 47 to 60 minutes were recorded and transcribed verbatim. The data were analyzed using qualitative content analysis as suggested by Graneheim and Lundman.

**Results:**

Four categories, 10 subcategories, and 25 condensed meaning units were identified by interpreting mothers’ underlying meanings. The four categories were ‘Knowledgeable yet contrasting ideas regarding EDCs,’ ‘Negative health impact, but more so for children,’ ‘Inaction or trying to minimize exposure,’ and ‘Need for early, reliable resources and social change.’ Mothers were knowledgeable regarding EDCs and actively needed further education and support. While they tended to focus more on the health impact of EDCs on their children and were optimistic about their health risks, paying less attention to their preventive behaviors.

**Conclusion:**

Healthcare professionals must consider mothers’ perceptions of EDCs in future education and interventions ensuring EDCs impact on women’s life stages such as puberty, pregnancy, and childrearing. Also preventive strategies that can be applied to their daily lives are needed.

## Introduction

Endocrine-disrupting chemicals (EDCs) are compounds that interfere with normal hormone function. They are primarily synthesized for industrial solvents, electronics, personal care products, plastics, and pesticides, and they include polychlorinated biphenyls, bisphenol A, phthalates, dioxins, DDT (dichlorodiphenyltrichloroethane), and others [1,2]. Since EDCs are ubiquitous in our living environment, their effects can be seen at all stages of life, including in developing fetuses, and persistent exposure to EDCs and the accumulation of EDCs in the human body can lead to health problems in children and adults [1,3]. Particularly, exposure to bisphenol A and polybrominated diphenyl ethers during pregnancy is associated with intrauterine growth restriction [4], higher levels of anxiety, depression, aggression, hyperactivity, and behavioral problems in children [5], and persistent lower cognitive abilities from the ages of 2 to 8 years [6], as well as lower attention levels and executive functioning at the ages of 9 to 12 years [7]. More importantly, constant exposure to EDCs in the environment can lead to the early onset of puberty and breast cancer [8], obesity, diabetes, thyroid dysfunction, and infertility [3,9]. Thus, it is critical to be aware of environmental hazards and the life-long health impacts of EDCs beginning in the early stages of life.

In this regard, parents are primarily responsible for protecting children from exposure to EDCs [10]. As children grow up, parents play multiple roles in the formation of their health behaviors by educating their children, acting as role models, and influencing their cognitive and behavioral characteristics [11]. Many studies have shown that parenting practices, particularly mothers’ parenting styles, influence their child(ren)'s body weight, diet, physical activity, and eating behaviors [12,13]. Due to the important role of mothers in families and social environments for protecting children from various harmful conditions, mothers should be engaged to and supported to guide children's health practices. Nevertheless, there is little research on the perception of EDCs among mothers, who tend to be children’s primary caregivers. Mothers are known, however, to share information about EDCs through social networking services [14]. It is important to understand mothers’ real experiences; what and how they are concerned about EDCs, what kind of matters they come across, and how they take care of it during childrearing. Therefore, a qualitative approach was considered appropriate for addressing the aims of this study exploring perspectives and ideas about EDCs among Korean mothers with young children, particularly as primary caregivers. This study aimed to explore the perceptions of EDCs among mothers with young children to provide a basis for parental education to enhance parents’ awareness concerning the risks of EDCs, particularly in a way that meets parents’ needs and expectations concerning childcare.

## Methods

**Ethical statement:** This study was approved by the institutional review board of Yonsei University Wonju Severance Christian Hospital (No. CR319012). Informed consent was obtained from the participants.

### Design

This study used an exploratory qualitative design to explore the perception of mothers with young children about EDCs for their concerns, the issues they faced, and the way they dealt with them. This study adhered to the COREQ (Consolidated Criteria for Reporting Qualitative Research) guidelines (https://www.equator-network.org/reporting-guidelines/coreq/).

### Participants

The participants were recruited from three locations in the Kangwon-do area selected through snowball sampling. A director of a local kindergarten was approached by the research team, and then the director as a key person introduced mothers to the study. Considering the distribution of childrens' age, two additional childcare centers were introduced by the key person, then each director of the center allowed the research team to recruit potential participants

The mothers were included in the study if they (1) were their child’s primary caregiver, (2) had at least one or more infants, toddlers, or preschoolers, and (3) provided written consent after agreeing to the purpose and processes of the study. There was no specific exclusion criterion for the mothers. Since mothers’ experiences could be influenced by the developmental processes of their children, we decided to explore various experiences of mothers so as not to be biased to a certain gender or age within the age criteria during the recruitment process.

The number of study participants in a qualitative study is considered sufficient once the threshold of reliable information is met, and the appropriate number of participants is generally assumed to be 1 to 30 [15]. A total of 12 mothers were interviewed and included in the final analysis as the data were saturated.

### Data collection procedure

For data collection, initial meetings with possible participants and the research assistant were arranged by the director of the kindergarten and childcare centers.

For mothers who agreed to participate, interviews were conducted by the principal investigator on a scheduled date and time in a private room in each institution. A research assistant with a master’s degree was also present in the room to record field notes about the interview environment and the non-verbal behaviors of the participants. Each participant was interviewed once and the each interview proceeded in a comfortable environment where the mothers could share their experiences in as much detail as possible. At the end of the interview, the mothers shared any questions or comments they had. A small gift (worth 25 US dollars) was given to the mothers to express gratitude for their time and cooperation.

All interviews were recorded, and the files were transcribed by the research assistant, using identification numbers to anonymize participants. Field notes were also utilized for the data analyses. Each interview lasted for approximately 47 to 60 minutes, with an average of 54 minutes.

### Interview questions

The four main questions were provided as follows; “Would you tell us what you know about EDCs?”, “What do you think about the impact of EDCs on you and your child(ren)?”, “What actions do you take to reduce the health impact of EDCs?”, and “What do you think are the most effective ways to reduce exposure to EDCs?”

### Data analysis

A total of 730 minutes of interviews was transcribed and analyzed according to Graneheim and Lundman’s [16] qualitative content analysis process method, which proceeded as follows: The researchers initially read the transcribed materials including field notes to get a sense of the whole, then started to mark meaningful phrases and sentences as units of analysis during the second reading. Then we selected meaning units separately and compared the outcomes to confirm the significant meaning units. Next, the selected meaning units responding to the study aim were reviewed and agreed upon regarding their core contents between the authors. Then condensed meaning units were composed and abstracted into subcategories and categories. During the analysis, we continuously discussed interpretations in all steps.

### Trustworthiness

To achieve trustworthiness, we tried to meet the credibility, confirmability, authenticity, dependability, and transferability criteria for qualitative studies [16]; to ensure credibility, we recruited mothers with young children of infants, toddlers, or preschoolers who were willing to share their experiences according to the aims of the study. In addition, recruitment was continued to include 12 mothers to obtain the richness and saturation of data. For confirmability and authenticity, the analyzed outcomes were verified by three mothers (each one from three age groups: an infant, a toddler, and a preschooler). They confirmed if the reports reflected mothers’ feelings, tones, expressions, and words of their experiences in richer and more vigorous ways. For dependability, we had more than five team meetings to ensure that no errors were made during the analysis, particularly, codes and supporting quotes from the original text were selected to be differentiated among subcategories and categories. In addition, we attempted to avoid any preconceptions and listened to the mothers’ experiences with a nonjudgmental attitude so that the participants would be able to openly express what they wanted to share. To ensure transferability, the selection criteria and characteristics of the mothers were described in the report to provide the content from which the findings could be transferred to other groups of mothers.

## Results

The general characteristics of the 12 mothers are presented in Table 1.

The average age was 34.3 years (range: 23–41 years), and the average number of children was 1.6 (range: 1–3). In terms of financial status, 11 mothers (91.7%) considered themselves middle-class, and six mothers (41.7%) were employed. Two mothers reported having thyroid disease and asthma, and there were four children diagnosed with precocius puberty or atopic dermatitis diagnosed with precocious puberty and atopic dermatitis, respectively.

A total of 288 major statements were identified in the interviews and after repeated reading of transcripts, 87 meaning units were found to be significant. A total of 25 condensed meaning units were then generated based on the interpretation of underlying meanings, which were further categorized into 10 subcategories and four categories. The mothers’ experiences of EDCs defined in the analysis were ‘Knowledgeable yet contrasting ideas regarding EDCs,’ ‘Negative health impact, but more so for children,’ ‘Inaction or trying to minimize exposure,’ and ‘Need for early, reliable resources and social change’ as shown in Table 2.

### Knowledgeable yet contrasting ideas regarding endocrine-disrupting chemicals

The mothers said that they knew EDCs as substances entering our body through various routes, in addition, some mothers expressed that EDCs were toxins accumulated in the bodies while others perceived EDCs as being harmless as they would decompose in the body.

#### Multiple routes of endocrine-disrupting chemicals entering the body

Five condensed meaning units were included in this subcategory. The mothers recognized that EDCs were substances that entered the body through various methods, such as using plastic containers or toys, heating food in disposable containers in the microwave, breathing in fumes from burning substances, and absorbing them through the skin from shampoo and cosmetics in daily life. In addition, they perceived EDCs as something that affected the fetus through the umbilical cord during pregnancy.

*"It’s in a lot of plastics… the biggest inflow would be through the mouth. Particularly, babies, they take everything to their mouth and suck their toys. I realize it strongly when I hear the news on TV about the detection of EDCs from toys.”* (participant 1)

*“I have heard from mom’s SNS. When we pour hot water in the white Styrofoam of cup noodles and when we put vinyl in the microwave... disposable containers for delivery food… those are all EDCs.”* (participant 5)

*“The smoky smell from something burned… that’s the EDCs. It also exists in the air and enters our body when we breathe.”* (participant 7)

*“It is also in the shampoos and cosmetics we use every day. It can infiltrate through our skin and eyes [laugh].”* (participant 8)

#### Contrasting ideas about endocrine-disrupting chemicals

In this subcategory, two condensed meaning units were explored. The mothers understood EDCs in a contrasting manner, with some viewing them as something accumulated in the body without being discharged and others believing that they are decomposed in the body.

*“Since EDCs are chemically generated, detrimental substances will be accumulated in our body if it is heated and transformed into EDCs. Because of environmental pollution, fruits grow deformed, so it is scary if the same thing could happen if EDCs are not eliminated from our body.”* (participant 9)

*“I’ve heard from my husband, a chemistry researcher that all those things accrue in women’s wombs. He always insists not to buy canned products, bleached toilet paper , etc..”* (participant 6)

*“I think we can be immune to EDCs as it is deformed like a virus… Maybe it could also be decomposed in our body if we follow a vegetarian diet.”* (participant 1)

*“How long are we going to live (laugh)… do people live forever if not eating fast foods, if not using cotton sanitary pads? A person who says EDCs are noxious seems to be picky. Eventually, we all die later. I don’t care much about that.” *(participant 11)

### Negative health impact, but more so for children

EDCs were believed to induce health problems in mothers and children were seen as more vulnerable to the effects of EDCs. However, some mothers were not concerned about the effects of EDCs on them or their children. They expressed different opinions on the influences of EDCs on health.

#### Causes of health problems for oneself

Three condensed meaning units were identified in this subcategory. Eating a large amount of instant food was a trigger for menstrual pain, and contact with EDCs caused allergies, asthma, and skin rashes. In addition, women felt the risk of infertility since they were born with a limited number of eggs, which could be affected by EDCs. The mothers acknowledged the relationship between EDCs and women’s reproductive health issues based on their experiences and allergic diseases associated with the environment.

*“I ate a lot of cup noodles and instant food when I was in high school and had worse cramping, which I never had before.” *(participant 6)

*“Unlike the first and the second pregnancy, I had trouble having a baby after... I think women would be more vulnerable to infertility than men because ovum is in women’s bodies from birth while sperm is constantly produced, isn’t it?” [nodding and looking at the interviewer]* (participant 2)

*“My asthma gets worse, too, when the air quality is bad, and wouldn’t it make the skin more sensitive?… and it won’t be good for those with weak bronchial tubes…”* (participant 4)

#### Higher vulnerability of children to the impact of endocrine-disrupting chemicals

Two condensed meaning units reflected the higher vulnerability of children rather than adults. The mothers expressed that their children had a higher chance of being exposed to EDCs as they are going to live in a developed, modern society for a longer time than we adults will live.

*“Kids are living in an environment with polluted air, vehicle smoke, and manufactured goods… so I think ADHD is increasing now…one of my friend’s kid was also diagnosed with it, kind of bad substances causing issues to the brain….”* (participant 12)

*“I see the difference clearly in my boy. Whenever he eats a lot of snacks, atopy symptoms are spreading under the genital area.” *(participant 7)

*“My daughter (8 years-old) is under treatment for precocious puberty, I found her breast budding one day. I blame myself; she might have had the disease because I gave her instant foods, cup noodles too much.” *(participant 3)

#### Optimism about one’s health problems due to endocrine-disrupting chemicals

In contrast, the mothers perceived that, since they did not currently have any particular health problems, any health issues from EDCs would be experienced in the distant future, if at all. In addition, they did not believe that adults would experience health issues from EDCs and felt they were far removed from such health problems.

*“I feel like I won’t have any problem due to EDCs in the short term. If it does, it will be about 20 to 30 years later… around menopause?”* (participant 9)

*“There is nothing I can directly feel. It’s invisible so it seems okay. To be honest, I don’t care…”* (participant 8)

### Inaction or trying to minimize exposure

For the question of what the mothers do to protect their health from EDCs, two opposing subcategories were identified. One position indicated keeping the same way of living because of the absence of disease and preference for a more convenient life. The other took actions to avoid exposure to EDCs based on the health problems of their children as well as their own health concerns.

#### Maintaining the easy ways of living

Two condensed meaning units were identified under this subcategory. Some felt no need for action since they were not currently experiencing any health problems, while others kept using convenient products despite the harms of EDCs. The mothers were aware of the destructiveness of EDCs, but they did not make any changes in their behavior for various reasons, such as a lack of current health issues, time management, and parenting convenience.

*“I think I’ll follow the advice only when I start feeling sick [laugh]. I have a habit of not doing something unless it is at hand.”* (participant 4)

*“It is hard to give up or to reduce the use of the microwave or an air fryer. I need those for timesaving when cooking for my baby or family [shamefaced smile].” *(participant 11)

#### Taking actions to reduce endocrine-disrupting chemicals exposure based on experiences

In this subcategory, two condensed meaning units were identified. The mothers tried to minimize their exposure to EDCs after their children experienced health problems and throughout pregnancy and childbirth. The mothers stated that they acknowledged the health problems of their children due to EDCs and that they took extra care to avoid EDCs that could harm the fetus during pregnancy.

*“My child has heavy atopic dermatitis, so I try to give as little instant food as possible and to give more homemade foods. It is hard to see him having a hard time because of itching so I try to do my best as a mother.” *(participant 3)

*“I started to be concerned when I got pregnant... so I was especially careful not to use the microwave and plastic containers. Now I wonder about the world our children will live in.” *(participant 10)

### Need for early, reliable resources and social change

The mothers expressed three subcategories related to their ideas for reducing their exposure to EDCs’ harms. The younger the child, the better the effects of education, so early education is essential. The mothers perceived that EDCs are associated with environmental pollution, which requires social agreement to establish policies. Finally, the mothers requested practical information for reducing EDC exposure.

#### Early education in children and its ability to spread

Two condensed meaning units were identified in this subcategory. The mothers believed early education about EDCs for children would be more effective for spreading information about EDCs and encouraging positive life habits. In addition, they believed that children could motivate their parents to change their behaviors by sharing what they learned.

*“I’m not interested in learning more about EDCs [quietly] but I think it will be beneficial to teach kids in the school curriculum because learning at a young age is most effective.”* (participant 4)

*“Once they learn, they will point out their moms’ faulty behavior. It will lead the parents to change their behavior… so I think it will be effective to educate the kids.”* (participant 6)

#### Social and systemic changes to reduce endocrine-disrupting chemicals

Under this subcategory, three condensed meaning units were identified. The mothers believed that government policy and regulation are needed due to the lack of awareness on reducing the consumption of products containing EDCs. In addition, they suggested continuous promotion through social media to raise awareness about EDCs. They also pointed out that environments where people can sell and buy eco-friendly products should be fostered.

*“People don’t seem to be urgent about EDCs’ risks and say ‘why now?’ That’s why I feel the need for some kind of governmental regulation.”* (participant 7)

*“I think societal awareness is important. Campaigns or social movements are what we need. Since social media is powerful today, information could spread through mom’s cafes or civic groups…”* (participant 11)

*“I hope prices are lowered with more movements for eco-friendly product consumption and with more production of them.”* (participant 10)

#### Reliable information applicable to daily life

Two condensed meaning units were identified related to this subcategory. The mothers wanted tangible information with scientific evidence to curtail the harms of EDCs, which are invisible toxic substances. Moreover, they wanted practical information that could be applied to their daily lives to reduce exposure to EDCs. They called for specific and realistic alternatives to motivate action.

*“Notable results of experiments on the impact of EDCs can be effective, like lung cancer photos on cigarette packs. That will be easier to understand, then we would be more careful, wouldn’t we?”* (participant 5)

*“When a person that I know gave me information about avoiding EDCs for cooking, it was useful for me to follow what I had heard.”* (participant 11)

*“Eradicating EDCs would be very unrealistic, so experts could recommend alternatives that are practical…”* (participant 1)

## Discussion

The study explored the perceptions about EDCs from the perspectives of mothers with young children using qualitative content analysis. Exposure to EDCs is known to occur through food, water, dermal contact, and inhalation [17], and the mothers in this study accurately recognized the transmission routes of EDCs. The mothers in this study, possibly due to their recent experiences of pregnancy and childrearing, were able to identify most of the possible routes of transmission, including the umbilical cord from the mother to the fetus. However, four of the mothers did not believe EDCs caused any bad consequences and decomposed gradually in the body. As suggested in prior literature [18] perceived EDCs as low risk since they are invisible and ubiquitous in daily life. As most of the mothers correctly perceived, EDCs accumulate and persist in organisms and the environment, and they may cause clinically observable and, when possible, measurable effects [19]. Thus, the long-term consequence of EDCs must be accurately shared with the public over time. Labeling consumer products with information would be a good way to increase risk perception, which has been observed to be effective in women of childbearing age [20]. As indicated in a previous study of 406 Korean adults [21], better knowledge about the environment would influence people to purchase more pro-environmental products.

The second distinct perception was about the relationship between EDCs and health problems. The mothers assumed that the vulnerability of children was much higher due to a higher chance of extended exposure to EDCs, which could potentially engage mothers to have more interest in preventive behaviors through future education and intervention. Therefore, it is important to provide information to reproductive aged women concerning the timing of exposure to EDCs, since developing fetuses and neonates are most vulnerable to endocrine disruption [22]. Indeed, many diseases and disorders are now considered to be related to prenatal exposure to EDCs, such as premature birth [23], autism [24], allergies [25], and congenital abnormalities [26]. While the mothers acknowledged various health problems due to EDCs, seven of the participants were optimistic that health problems due to EDCs would only happen to them in the distant future. Although EDCs have a very long half-life in the body and their adverse effects manifest at later ages [17], it is still important for parents to maintain a healthy lifestyle to prevent diseases and to be role models for their children. Above all, optimism hinders people from engaging in preventive health behaviors [27,28]. Thus, strategies need to be devised to minimize inaccurately optimistic views and increase awareness about the potential health consequences of EDC exposure.

The extent to which the mothers in this study understood the health impacts of EDCs appeared to correspond to their proactive behaviors to avoid the harms of EDCs. The mothers’ actions to avoid exposure to EDCs mostly related to their children’s health problems, even at the time of pregnancy and childbirth, rather than their own health issues. However, the mothers tended to maintain their lifestyles due to the convenience of using products with EDCs, which was similar to the findings of a study about the attitudes of young adult women in Korea concerning EDCs [29]. Mothers of young children are generationally accustomed to a high-convenience lifestyle and tend to consume more fast foods or processed foods and disposable products. Unconscious exposure to EDCs occurs through various routes of transmission. In particular, dietary intake accounts for more than 90% of total exposure to EDCs [30]. Therefore, mothers must understand their own vulnerability and health risks related to EDCs and ultimately make sustainable behavioral changes, especially as the primary caregiver of their child(ren) [31].

One of the main categories from the interviews with the mothers was that they required various ways to reduce their exposure to EDCs, such as actions based on the precautionary principle, regulatory actions, scientific evidence, and changes in the awareness of the general population about EDCs on social media. These varied demands indicate that detailed and concrete strategies that are applicable at the individual, public, and government levels are needed. Media, which comprise a major source of information, are an important determinant of risk perception [18,32] Media is noteworthy, not only to ensure that the public maintains a positive attitude toward precautionary measures [33] but also as an easy and effective way to guide the public to improve their knowledge, perception, and behaviors [34,35]. Therefore, media strategies are needed to disseminate how EDCs evoke negative hormonal mechanisms in the human body and how behavioral changes could lead to positive changes to protect people from the harms of EDCs.

Our study also found a strong need for early education about EDCs for children to establish positive life habits to prevent EDC exposure. The mothers anticipated more significant educational effects in their children rather than in themselves and expected their children to influence themselves. Life habits developed in early childhood affect the way individuals observe and respond to others’ health habits and can influence that individual’s family members to acquire knowledge, skills, and attitudes to improve their health [36]. A previous study found that children aged 4 to 5 years who had learned about passive smoking prevention insisted that their family members quit smoking [37]. Thus, environmental education during early childhood would be an effective strategy for influencing children to grow up with good health habits and also inspire behavioral changes in their parents. Information technology such as virtual reality would be a suitable mode to raise awareness of EDCs as well as to reduce exposure to EDCs.

It is possible that mothers with more interest in EDCs could have possibly participated in the study due to their own or their children’s health problems such as asthma, atopic dermatitis, or precocious puberty. Thus, it could be a possible bias and future studies should explore a wider range of perceptions of mothers.

In conclusion, mothers in this study were knowledgeable about EDCs and actively needed further education and support. While they tended to focus more on the health impact of EDCs on their children and were optimistic about their health risks, paying less attention to their preventive behaviors.

As strategies to prevent exposure to EDCs are urgently needed, prenatal education programs could include the topic of EDC exposure and its health impacts, particularly the routes of exposure not only to the mother but to the fetus. Also, parents and teachers need to be prepared in advance with accurate information and preventive strategies, to enable them to guide their young children to apply awareness, knowledge, and preventive behavioral habits in their daily lives. Overall, education programs to improve the publics general understanding of EDCs and the consequences of EDC exposure for students, health professionals (especially in endocrinology, pediatric, and maternity clinics) and laypersons are needed. Healthcare professionals should also provide the public with practical guidelines for health behaviors related to EDCs. It is also important to keep in mind that this requires political will to limit the use of these chemicals and develop and implement remediation technologies.

## Figures and Tables

**Table 1.** Demographic characteristics of the mothers and their children

Participant’s ID	Age (yr)	Educational status	Employment	Perceived economic status	Numbers, sex, and ages of children	Current health problem
1	23	College	No	Middle	1 boy: 9 months	
2	33	High school	No	Low	2 girls: 7 and 3 years	
					1 boy: 11 months	
3	35	High school	No	Middle	1 girl: 8 years	Daughter: precocious puberty
					1 boy: 4 years	Son: atopic dermatitis
4	40	College	Employed	Middle	1 girl: 12 years	Mother: asthma
					1 boy: 5 years	
5	32	College	No	Middle	1 girl: 2 years	
6	33	College	Employed	Middle	1 boy: 3 years	
7	41	College	Employed	Middle	2 boys: 12 and 8 years	Mother: thyroid disease
					1 girl: 5 years	Two sons: atopic dermatitis
8	34	High school	Employed	Middle	1 girl: 7 years	
					1 boy: 3 years	
9	32	High school	No	Middle	1 boy: 2 years	
10	33	College	No	Middle	1 girl: 4 years	
11	35	College	Employed	Middle	2 boys: 5 and 3 years	
12	40	College	Employed	Middle	1 boy: 9 years	
					1 girl: 5 months	

**Table 2.** Experiences of endocrine-disrupting chemicals (EDCs) among mothers with young children

Condensed meaning unit	Subcategory	Category
Chemicals that can enter through the mouth when using products made of plastics	Multiple routes of EDCs entering the body	Knowledgeable yet contrasting ideas regarding EDCs
Chemicals that can be delivered from the mother to the fetus through the umbilical cord		
		
Chemicals produced when using disposable food containers in the microwave		
Chemicals that can be breathed in from something burning		
Chemicals that can be absorbed through the skin from products		
Toxins that accumulate in the body	Contrasting ideas about EDCs	
Substances that are not harmful and are decomposed in the body		
Menstrual pain triggered via the consumption of instant foods	Causes of health problems for oneself	Negative health impact, but more so for children
Allergy, asthma, or skin rash via contact with EDCs		
Greater susceptibility to infertility in women because ova are already inside the body unlike sperm		
Children living in a developed society with ubiquitous sources of EDC exposure	Higher vulnerability of children to the impact of EDCs	
EDC exposure as dangerous for children due to longer-term and more frequent exposure		
Not a pressing issue to me	Optimism about one’s health problems due to EDCs	
No sense of active harm to myself from EDCs		
No need for action because of the absence of current health issues	Maintaining the easy ways of living	Inaction or trying to minimize exposure
Continuing to use convenient products despite the harms of EDCs		
Minimizing my exposure to EDCs after my children experienced health problems	Taking actions to reduce EDC exposure based on experiences	
Trying to avoid exposure to EDCs throughout pregnancy and childbirth		
Early education for children for better effects	Early education in children and its ability to spread	Need for early, reliable resources and social change
Parental changes via the influence of children		
Establishment of policies and regulations to reduce EDCs	Social and systemic changes to reduce EDCs	
Continuous public awareness through social media		
Environment for adopting eco-friendly products		
Understandable information backed by evidence	Reliable information applicable to daily life	
Practical information applicable to daily life		
